# HIF-1α promotes the migration and invasion of cancer-associated fibroblasts by miR-210

**DOI:** 10.14336/AD.2021.0315

**Published:** 2021-10-01

**Authors:** Ying Yang, Junjie Gu, Xuechun Li, Chunling Xue, Li Ba, Yang Gao, Jianfeng Zhou, Chunmei Bai, Zhao Sun, Robert Chunhua Zhao

**Affiliations:** ^1^Department of Medical Oncology, Peking Union Medical College Hospital, Chinese Academy of Medical Sciences and Peking Union Medical College, Beijing 100730, China.; ^2^Institute of Basic Medical Sciences Chinese Academy of Medical Sciences, School of Basic Medicine Peking Union Medical College, Peking Union Medical College Hospital, Center of Excellence in Tissue Engineering Chinese Academy of Medical Sciences, Beijing Key Laboratory (No. BZO381), Beijing 100005, China

**Keywords:** cancer-associated fibroblasts, migration, invasion, colorectal cancer, HIF-1α

## Abstract

Metastasis is the major cause of death in colorectal cancer (CRC) patients. Inhibition of metastasis will prolong the survival of patients with CRC. Cancer cells bring their own soil, cancer-associated fibroblasts (CAFs), to metastasize together, promoting the survival and colonization of circulating cancer cells. However, the mechanism by which CAFs metastasize remains unclear. In this study, CAFs were derived from adipose mesenchymal stem cells (MSCs) after co-culture with CRC cell lines. Transwell assays showed that CAFs have stronger migration and invasion abilities than MSCs. In a nude mouse subcutaneous xenograft model, CAFs metastasized from the primary tumour to the lung and promoted the formation of CRC metastases. The expression of HIF-1α was upregulated when MSCs differentiated into CAFs. Inhibition of HIF-1α expression inhibited the migration and invasion of CAFs. Western blot and ChIP assays were used to identify the genes regulated by HIF-1α. HIF-1α regulated the migration and invasion of CAFs by upregulating miR-210 transcription. Bioinformatics analysis and luciferase reporter assays revealed that miR-210 specifically targeted the 3’UTR of VMP1 and regulated its expression. Downregulation of VMP1 enhanced the migration and invasion of CAFs. In vivo, inhibition of miR-210 expression in CAFs reduced the metastasis of CAFs and tumour cells. Therefore, the HIF-1α/miR-210/VMP1 pathway might regulate the migration and invasion of CAFs in CRC. Inhibition of CAF metastasis might reduce CRC metastasis.

Colorectal cancer (CRC) is one of the most common malignancies in the world [1]. Metastasis is the main cause of death in patients with CRC, and inhibition of metastasis prolongs the survival of CRC patients.

Cancer-associated fibroblasts (CAFs) are the most abundant non-malignant cells in the tumour microenvironment [2], and they might play an important role in promoting the metastasis of tumour cells from the primary site and colonization in distant organs. CAFs induce the Epithelial-mesenchymal transition (EMT) of cancer cells through paracrine signalling with growth factors and pro-migratory cytokines [3]. Transforming growth factor-β (TGF-β) and hepatocyte growth factor (HGF) derived from CAFs promote the EMT and metastasis of cancer cells [4, 5]. CAFs secrete high levels of chemokine ligand 7 and chemokine ligand 16, which promote both the migration and invasion of hepatocellular carcinoma cells by enhancing the activity of the TGF-β pathway [6]. Calvo et al. revealed that CAFs mediated tumour microenvironment (TME) remodelling and enhanced extracellular matrix (ECM) stiffening to facilitate tumour cell invasion by activating the transcription factor YAP1 and regulating cytoskeletal proteins [7]. CAFs can also reshape the basement membrane, leading to the formation of gaps through which cancer cells can migrate [8]. The interaction between N-cadherin on CAFs and E-cadherin on tumour cells drives tumour cells invasion dynamically [9]. In addition, as an important component of the TME, CAFs inhibit apoptosis and promote matrix proliferation and angiogenesis to establish a metastatic microenvironment to facilitate tumour cell colonization [10, 11].

The origin of CAFs in metastatic sites remains controversial. Most studies have suggested that CAFs in metastatic sites are derived from intrinsic fibroblasts of the metastatic organ [12, 13]. However, Duda et al [14] found that metastatic cells could bring stromal components, including CAFs, from the primary site to the lung, and these co-travelling CAFs provided an early growth advantage to the metastatic cancer cells in the lung. Furthermore, recent studies have found the presence of circulating CAFs (cCAFs) in the peripheral blood of patients with a variety of tumours [15, 16]. Based on these results, we hypothesize that CAFs may help to form a microenvironment for tumour cell colonization. However, the mechanism by which CAFs metastasize remains unclear.

Since normal MSCs from circulating or adjacent tissues are one of the main sources of CAFs [17-19], we established a model in which colorectal cancer cells induced MSCs to become CAFs. The migration and invasion abilities of CAFs were significantly higher than those of MSCs. HIF-1α was significantly upregulated during the differentiation of MSCs into CAFs, and the inhibition of HIF-1α significantly inhibited the migration and invasion of CAFs. We then planned to study the mechanism by which HIF-1α regulates the migration and invasion of CAFs and whether inhibiting CAF metastasis could reduce tumor metastasis.

## MATERIALS AND METHODS

### Isolation and culture of human adipose-derived MSCs

Isolation and culture of MSCs were performed in accordance with the Declaration of Helsinki and approved by the Institute of Basic Medical Sciences, Chinese Academy of Medical Sciences (Project No. 022-2015) [20, 21]. Human adipose tissue was obtained from patients undergoing tumescent liposuction approved by orthopaedic hospitals, and all samples were used with informed consent from donors. Adipose tissue-derived mesenchymal stem cell culture was performed as described previously [21].

### Tumor cell culture

The colorectal cancer cell lines HCT-8, HCT-116 and LOVO were obtained from the Cell Resource Center, IBMS, CAMS/PUMC (Beijing, China). All of the cell lines were cultured in high-glucose Dulbecco’s modified Eagle’s medium (H-DMEM) supplemented with 10% foetal bovine serum (Gibco, Carlsbad, CA, USA) and were incubated at 37 ? in a 5% CO_2_ humidified incubator. After 2 or 3 days, the cells were digested and passaged.

### Co-culture

A Transwell chamber with a 0.4 μm pore size permeable membrane (Cat. #3450, Corning, Corning, USA) was used to co-culture MSCs with colorectal cancer cells (HCT-8, HCT-116 and LOVO). The MSCs were plated in the lower chamber of a six-well transwell plate at a density of 2 × 10^5^/ml, and colorectal cancer cells (2 × 10^5^/ml) were plated in the upper chamber. All cells were incubated at 37 °C in a 5% CO_2_ humidified incubator. After 7 day of co-culture, the MSCs could be induced to CAFs for subsequent experiments.

To avoid the effect of the HIF-1α selective protein inhibitor on tumor cells during co-culture, conditioned medium was used instead of co-culture. The culture medium of colorectal cancer cells after 24 h of culture was collected as the conditioned medium for the following experiment. Previous studies suggested that the effect of conditioned medium was similar to the effect of direct co-culture [22, 23].

### Adipogenic differentiation

Adipogenic differentiation was performed as previously described [24]. The culture medium was replaced with adipogenic medium composed of H-DMEM supplemented with 10% foetal bovine serum, 1 μm dexamethasone (Sigma-Aldrich, St. Louis, MO, USA), 0.5 mm isobutylmethylxanthine (Sigma-Aldrich, St. Louis, MO, USA) and 1 mm L-ascorbic acid (Sigma-Aldrich, St. Louis, MO, USA). After 12 days, the cells were washed with PBS and stained with filtered oil red O solution (Sigma-Aldrich, St. Louis, MO, USA). Then, positive cells were quantified under a microscope.

### Osteogenic differentiation

Osteogenic differentiation was performed as previously described [24]. The cells were induced in osteogenic medium composed of high-glucose Dulbecco’s modified Eagle’s medium (H-DMEM) supplemented with 10% fetal bovine serum, 10 mm β-glycerophosphate (Sigma-Aldrich, St. Louis, MO, USA), 0.5 mm L-ascorbic acid and 0.01 mm dexamethasone. After 5 days, alkaline phosphatase (ALP) staining was performed using an ALP staining kit (Institute of Hematology and Blood Diseases Hospital, Chinese Academy of Medical Sciences, Tianjin, China) according to the manufacturer’s protocol. Alizarin red staining was performed to detect matrix mineralization deposition during the later stage of osteogenesis.

### RNA extraction

RNA extraction and qRT-PCR were performed as described previously [25]. The culture medium was discarded, and 1 ml of TRIzol was added to 1×10^6^ cells at room temperature for 5 min. The lysate was moved to an enzyme-free EP tube and stored at -80 ? or used immediately to extract RNA. Chloroform (200 μl) was added to each tube, which was shaken vigorously for 30 s and mixed at room temperature for 5 min. After centrifugation at 12,000 rpm for 15 min at 4 ?, the upper layer was transferred to a new enzyme-free EP tube. An equal volume of isopropanol was added, the tube was inverted gently, and the sample was incubated at room temperature for 10?min. After incubation, the sample was centrifuged at 4 °C at 12,000?rpm for 15?min, and the supernatant was discarded. One millilitre of prechilled 75% ethanol [dehydrated ethanol plus enzyme-free diethyl pyrocarbonate (DEPC) water] was added to clean the sediment (without disrupting the pellet), and then the sample was centrifuged at 7,500?rpm for 5?min. This step was repeated once, and the supernatant was discarded. The sample was air-dried until the precipitate was clear. Thirty microlitres of DEPC water was added to dissolve the RNA precipitate, and a Nanodrop spectrophotometer (Thermo Scientific NanoDrop 2000/2000C) was used to measure RNA concentrations.

### qRT-PCR analysis

cDNA was reverse transcribed (30?μl), and experiments were performed following the procedure recommended by TaKaRa M-MLV reverse transcriptase (Takara Bio, Inc.) product specifications. Quantitative reverse transcription-polymerase chain reaction (qRT-PCR) experiments were performed according to the recommended methods provided by the manufacturer of the TaKaRa SYBR® Premix Ex Taq kit (Takara Bio, Inc.). U6 and GAPDH were used as housekeeping genes for standardization. The primer sequences are listed in Supplementary Table 1. Data are presented as the fold change of downregulation or upregulation (fold value = 2^-ΔΔCt^, where ΔΔCt = (Ct of the gene of interest, treated-Ct of the housekeeping gene, treated) - (Ct of the gene of interest, control-Ct of the housekeeping gene, control), and Ct was the number of threshold cycles).

### Western blot assay

Western blot assays were performed as previously described ^[21]^. The culture medium was discarded. The cells were washed with prechilled PBS, and neutral Radio Immunoprecipitation Assay lysate containing 1 mm phenylmethylsulfonyl fluoride was added. Then, the cells were lysed on ice for 10 min and manually scraped from the culture plates into a 1.5 ml EP tube. After centrifugation at 12,000 rpm for 30 min, the supernatant was transferred to a new EP tube. The cells were divided into two parts: one part was frozen at -20 °C, and the other part was used to determine the concentration. The protein concentration was measured with the BCA protein concentration measurement kit (P0011; Beyotime). To the other part, 5 × protein electrophoresis loading buffer mix was added at a 1:4 ratio, and the mixture was boiled in a boiling water bath (5-10 min). Fifteen micrograms of samples per lane were loaded onto an SDS-PAGE (sodium dodecyl sulphate polyacrylamide gel electrophoresis) protein gel for protein separation. Polyvinylidene difluoride (PVDF) membranes were used for wet transfer. A 5% skim milk solution was used to block the hybridization reaction. Proteins were incubated with the indicated primary antibody, followed by incubation with the secondary antibody. The antibodies used were as follows: HIF-1α rabbit mAb (1/1000; Cat. #14179, Cell Signaling Technology, Danvers, MA, USA), TMEM49/VMP1 rabbit mAb (1/1000; Cat. #12929, Cell Signaling Technology, Danvers, MA, USA), alpha SMA-specific rabbit mAb (1/1000; Cat. # 55135-1-AP, ProteinTech, Chicago, IL, USA), FAPA rabbit mAb (1/1000; Cat. # 66562, Cell Signaling Technology, Danvers, MA, USA), GAPDH rabbit mAb (1/1000; Cat. # sc-20358, Santa Cruz Biotechnology, Santa Cruz, CA, USA) and Lamin B1 rabbit mAb (1/1000; Cat. #13435, Cell Signaling Technology, Danvers, MA, USA). The secondary antibody used was the anti-rabbit horseradish peroxidase-conjugated antibody (1/3000; Santa Cruz Biotechnology). Antibody-antigen complexes were detected using ECL reagent (Millipore Corporation, Billerica, MA, USA).

### Cell migration and invasion assays

Cell migration and invasion assays were performed as previously described [26]. Transwell chambers (8 μm pore size; Costar, Kennebunk, ME, USA) were used in the migration and invasion assays. CAFs or MSCs were seeded into the upper well without FBS. The lower well was filled with 10% FBS as the invasion or migration attractant. Chamber membranes were coated with Matrigel for the invasion assay. After 36-48 h, the cell culture solution was discarded. Then, the upper well was washed with PBS, fixed with 4% paraformaldehyde for 10 min, and stained with crystal violet. Finally, the migrating or invading cells were counted and photographed.

### Lentiviral transduction particle preparation and transduction

This protocol was performed as previously described [27]. The miR-210 mimics, Si210 and a negative control (NC) expression cassette were first constructed in LV10 cells (U6/mCherry & Puro) (GenePharma, Suzhou, China). An NC expression cassette was also constructed in LV3 (H1/GFP & Puro) and LV16 (U6/mCherry-Luciferase17 & Puro) (GenePharma, Suzhou, China) cells. The inserted sequences were as follows: Si210: 5’-TCAGCCG CTGTCACACGCACAG-3’, NC: 5’-CAGUACUUUG UGUAGUACAA-3’, and miR-210 mimics: 5’-CTGTG CGTG TGACAGCGGCTGA-3’. Lentivirus was added to the cell culture medium according to the manufacturer’s instructions. Culture medium containing puromycin (2 μg/ml) was used to select cells 24 h after transduction. The transduction efficiency was evaluated by detecting mCherry/GFP expression under a fluorescence microscope. Transfection efficiency was then verified by qRT-PCR.

### ChIP assay

Chromatin immunoprecipitation (ChIP) was performed using an EZ-ChIP™ Chromatin Immunoprecipitation Kit (Millipore, Germany, 17-371) according to the manufacturer’s protocol. The control group and co-culture group were cross-linked with 1% formaldehyde for 10 min and prepared for ChIP. The immune-precipitated DNA was detected by electrophoresis. We used the following antibody in the ChIP procedure: anti-HIF-1α rabbit mAb (CST, D2U3T). Normal rabbit IgG was used as a negative control. The primer set was chosen to amplify approximately 500-600 bp around the indicated region. The primers of the promoter were as follows: miR-210-promoter-F: 5’-ATTCTCGAG GGCGGGAGGAGGACCACCTC-3’; and miR-210-promoter-R: 3’-AATAAGCTTGGGCGGGCGGAGGG ATTGAC-5’.

### Dual-luciferase reporter assay

The 3’ untranslated region (UTR) of the wild-type (wt) human VMP1 gene, which included the putative hsa-miR-210 binding site, was inserted into the dual-luciferase pmirGLO vector (GenePharma, Suzhou, China). The other gene which included the mutated (mut) hsa-miR-210 binding site, was also inserted into the pmirGLO vector. 293T cells were seeded into 6-well plates and transfected with luciferase vectors containing VMP1 (wt), VMP1 (mut), a negative control (NC) or a positive control (PC). Forty-eight hours after transfection, relative luciferase activities were measured using a dual luciferase reporter assay (Promega, USA) according to the manufacturer’s instructions.

### Rescue assay

The plasmid vector of the VMP1 expression clone was purchased from GenePharma (GenePharma, Suzhou, China). The VMP1 overexpression or control plasmid was transfected into control-MSC/miR-210 mimics-MSCs with Lipofectamine 2000 (Invitrogen, Carlsbad, CA, USA). The transfection efficacy was determined by qRT-PCR at 24 h, and cell migration and invasion assays were performed at 36-48 h.

### Flow cytometry assay

According to the reported method of flow cytometry assay [21], samples were added into the EP tube and centrifuged at 1,200 rpm for 5 min. A total of 200 μl of a 1:100 dilution of PE mouse anti-human antibodies (CD106, CD34, CD44, CD31, CD29, and CD90) (BD Pharmingen, La Jolla, CA) were added to each tube and mixed. The same-species, same-isotype irrelevant antibody was used as negative control. Samples were incubated at 4 ? for 30 min, and then centrifuged at 1,200 rpm for 5 min. The supernatant was discarded. The sediment was mixed with 200 μl of PBS. Cells were analyzed using the BD Accuri C6.

### Adhesion assay

According to the reported method of adhesion assay [26], 100 μg/ml fibronectin (FN) was added to the 96-well culture plate. After incubated overnight at 4 °C, the plate was washed with PBS and blocked with 1% BSA. Then the coated plate was washed twice with H-DMEM and used for the adhesion assay. The BSA control group was not coated with FN and treated with 1% BSA alone. Cells were resuspended at a concentration of 0.5 × 10^6^ cells / ml and added to FN-coated culture plate and BSA control culture plate at 100 μl / well. After incubated for 60 min at 37 °C, the plates were washed with PBS. The attached cells were fixed with 4% paraformaldehyde, stained with crystal violet, dissolved with glacial acetic acid, and detected with a plate reader at 570 nm. The number of adherent cells was OD of the FN-coated group minus that of the BSA control group.

**Figure 1.**
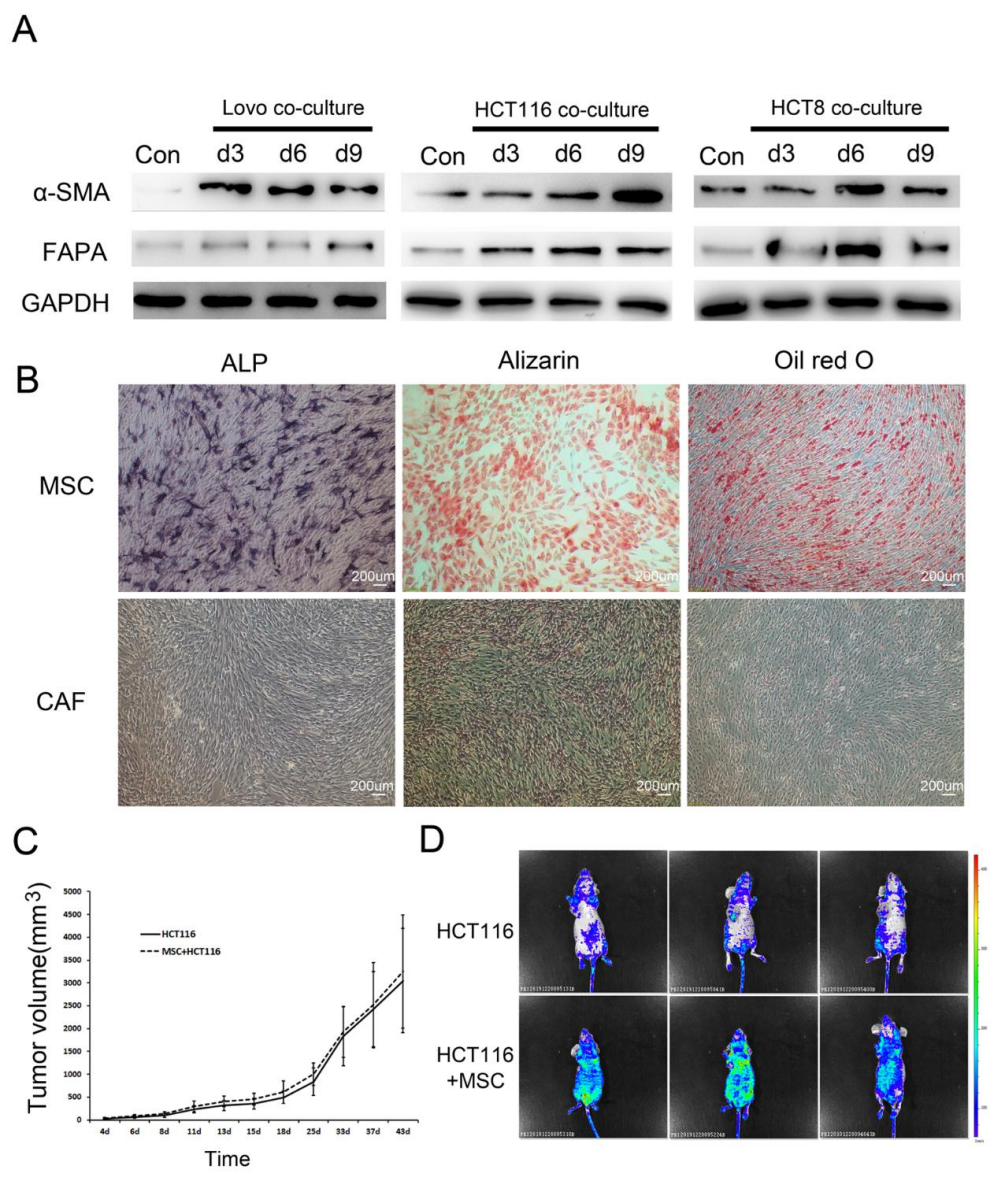
MSCs were induced to CAFs by colorectal cancer cells. (A) Western blot showing that the expression of the CAF characteristic proteins α-SMA and FAPA was upregulated after MSCs were co-cultured with LOVO, HCT-116, and HCT-8 cells. (B) Comparison of adipogenic (Oil Red O staining) and osteogenic (alizarin red and alkaline phosphatase staining) differentiation abilities between MSCs and CAFs. Oil Red O staining and alizarin red staining were performed after 12 days of culture. Alkaline phosphatase staining was performed after 5 days of culture. (C) Effect of combined MSC transplantation on primary tumour volume in nude mice. HCT-116 group: HCT-116 5 × 10^6^ cells; MSC + HCT-116 group: HCT-116 5 × 10^6^ cells + MSC 1 × 10^6^ cells. (D) In vivo fluorescence image showing the effect of combined MSC transplantation on tumour metastasis. Two groups of mice were transplanted with HCT-116 5 × 10^6^ cells and HCT-116 5 × 10^6^ cells + MSC 1 × 10^6^ cells. The HCT-116 cell lines carried GFP.

### Animal experiments

Animal experiments were performed as previously described [26]. All male nude mice (BALB/c-nu) were six to eight weeks old and were purchased from the Experimental Animal Institute of the Chinese Academy of Medical Sciences (Beijing, China). All mice were bred and maintained under specific pathogen-free (SPF) conditions in individually ventilated (high-efficiency particle-arresting filtered air) sterile microisolator cages (Techniplast, Milan, Italy). The experimental procedures were approved by the Animal Care and Use Committee of the Chinese Academy of Medical Sciences.

For the differentiation of MSCs into CAFs (metastasis-promoting assay), HCT-116 cells were stably transduced with lentivirus vectors (GFP). MSCs were also stably transduced with lentivirus vectors (mCherry). For the HCT-116 group (n=5), HCT-116 cells were resuspended in PBS at a concentration of 5 × 10^6^/100 μl. Male BALB/c nude mice (5-6 weeks old) were then injected with 100 μl of cell suspension subcutaneously. For the HCT-116+MSC group (n=5), HCT-116 cells were injected with MSCs at a ratio of 5:1. The tumor sizes were measured every week, and the volumes (in cubic millimetres) were calculated according to the following equation: width^2^ × length × 0.5. In vivo fluorescence imaging was used to detect HCT-116 cell metastasis.

For the CAF metastasis assay, MSCs were stably transduced with lentivirus vectors (mCherry-luciferase). For the HCT-116 group (n=5), HCT-116 cells were also injected subcutaneously. For the HCT-116+MSC group (n=5), HCT-116 cells were injected with MSCs (mCherry-luciferase). After 8 to 9 weeks, in vivo fluorescence imaging detected CAF metastasis. Mice underwent histological evaluations of lung metastasis.

To analyze the role of miR-210 in CAFs and tumor metastasis, MSCs in which miR-210 expression was altered and the corresponding controls (stably transduced with the mCherry lentivirus vector) were mixed with HCT-116 cells at a ratio of 1:5. For the HCT-116 group (n=10), HCT-116 cells were also injected subcutaneously. For the HCT-116+Si-miR-210-MSC group (n=10)/Si-control-MSC group (n=10), HCT-116 cells were injected with Si-miR-210-MSCs/Si-control-MSCs. The area of mCherry^+^ cells was detected by ImageJ.

**Figure 2.**
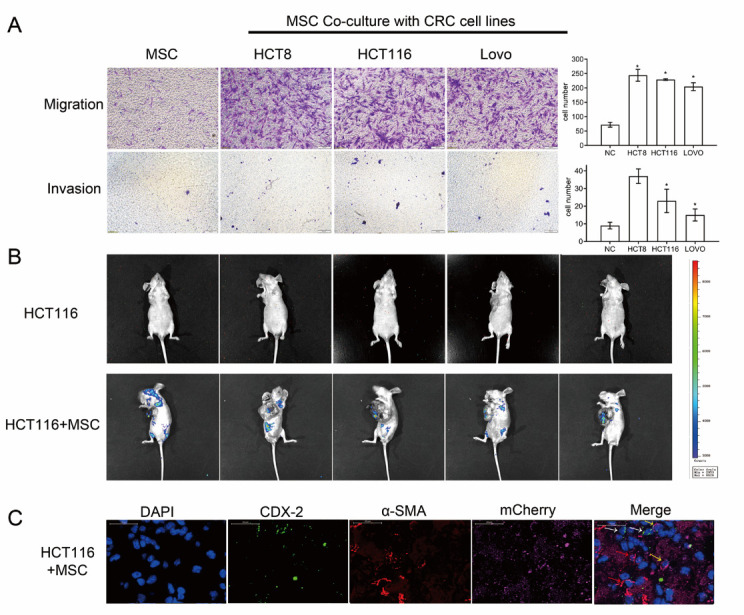
CAFs had stronger migration and invasion abilities than MSCs. (A) Transwell assays (n = 4) showed that CAFs had stronger migration and invasion abilities than MSCs. * *P* < 0.05. Scale bar: 200μm. (B) In vivo fluorescence image showing CAFs expressing luciferase in parts other than the primary foci in the HCT-116 + MSC group of the nude mouse subcutaneous transplantation model. Lentivirus vectors carrying mCherry-luciferase were transfected into MSCs. HCT-116 5 × 10^6^ cells and HCT-116 5 × 10^6^ + MSC 1 × 10^6^ cells were transplanted into the two groups. (C) Multiple immunofluorescence staining showing colorectal cancer cells and CAFs originating from the primary tumour in the lung metastases in mice of the HCT-116 + MSC group. DAPI: nucleus (blue), CDX-2: colorectal cancer cells (green), α-SMA: CAFs (red), mCherry: exogenous CAFs (violet). White arrows: CDX-2-positive cells (CRC cells), yellow arrows: both α-SMA- and mCherry-positive cells (exogenous CAFs), red arrow: only α-SMA-positive cells (endogenous CAFs). Scale bar: 20μm.

### Statistical analysis

All data are expressed as the mean ± standard deviation, and two-tailed t-tests and one-way ANOVA (analysis of variance) were performed. *P*<0.05 was considered significant. Each experiment was repeated at least three times to obtain a *P* value and to control for systematic errors.

## RESULTS

### CRC cell lines induced the differentiation of MSCs into CAFs.

Previous studies have found that MSCs can home in cancer and differentiate into CAFs in vivo [28]. To study the functions of CAFs derived from MSCs in CRC, we first determined whether MSCs could differentiate into CAFs after co-culture with the colorectal cancer cell lines LOVO, HCT-116 and HCT-8 in vitro. The results showed that the expression of α-SMA and FAPA was significantly upregulated after MSCs were co-cultured with CRC cells (Fig. 1A, Supplementary Fig. 1). In addition, the multiline differentiation ability of MSCs disappeared after co-culture (Fig. 1B). Further analysis of the cell mesenchymal marker of CAFs and MSCs indicated that the positive rate of CD44, CD90, and CD29 was more than 90%, and CD31, CD 34, and CD 106 was negative (Supplementary Fig. 2A). The result showed that CAFs have the same mesenchymal markers as MSCs. The expression of IL-8, IL-10, MCP-1, HGF in CAFs was significantly higher than that in MSCs (*p*<0.01, Supplementary Fig. 2B). The adhesion ability of CAFs was significantly lower than that of MSCs (*p*<0.01, Supplementary Fig. 2C). These results suggested that MSCs could be induced to differentiate into CAFs by colorectal cancer cells.

**Figure 3.**
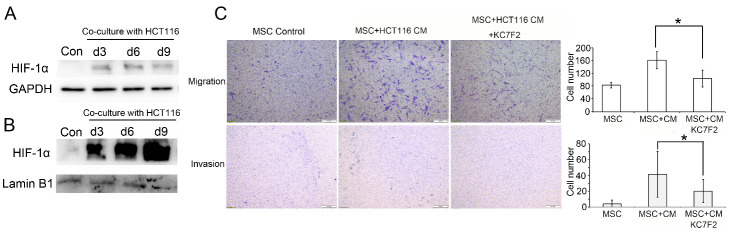
HIF-1α regulated the migration and invasion of CAFs. (A) Western blot assay of HIF-1α protein levels during the differentiation of MSCs into CAFs induced by HCT-116 cells. (B) Western blot assay of HIF-1α protein levels in the nucleus during the differentiation of mesenchymal stem cells (MSCs) into CAFs induced by HCT-116 cells. (C) Transwell assays (n = 4) showing that the migration and invasion capacities of CAFs were significantly decreased after treatment with the HIF-1α inhibitor KC7F2. * *P* < 0.05. Scale bar: 200 μm.

We further transplanted HCT-116 or HCT-116 cells combined with MSCs into nude mice to establish a subcutaneously implanted tumour model (HCT-116 cells were transfected with GFP, and MSCs were transfected with mCherry). It was indicated that exogenous MSCs could differentiate into CAFs that expressed both mCherry and α-SMA in the subcutaneously transplanted tumors of the HCT-116+MSC group (Supplementary Fig. 3). There was no significant difference in the volumes of subcutaneously transplanted tumors between the HCT-116 and HCT-116+MSC groups (Fig. 1C). However, in vivo fluorescence imaging revealed that the HCT-116+MSC group exhibited significantly increased metastasis (Fig. 1D), suggesting that one of the main functions of CAFs is to promote tumor metastasis in CRC.

### CAFs have migration and invasion abilities

cCAFs have been reported in the peripheral blood of patients with CRC [15]. We then examined the mechanism by which CAFs metastasize into peripheral blood vessels.

First, the migration and invasion capacities of MSCs and CAFs were analyzed via transwell assays. The results showed that MSCs had some migration capacity, but CAFs had significantly higher migration capacity than MSCs. In addition, MSCs had almost no invasive capacity, but a small number of cells showed invasion capacity after differentiating into CAFs (Fig. 2A). We then examined whether the CAFs of the primary tumor could metastasize to other body parts in the nude mouse subcutaneous transplantation model. We transfected lentivirus vectors carrying mCherry-luciferase into MSCs. In the above experiments, we confirmed that MSCs could differentiate into CAFs in the subcutaneous primary tumor (Supplementary Fig. 3). After 8-10 weeks of subcutaneous transplantation, the in vivo fluorescence imaging system detected luciferase in sites other than the primary foci in the HCT-116+MSC group, which indicated that CAFs carrying luciferase could metastasize from the primary tumor to other body parts (Fig. 2B).

Then, multiple immunofluorescence staining was used to detect CRC cells and CAFs in the lung metastases of mice. CDX-2 is a transcription factor specific to CRC. Colorectal cancer cells were detected by tagging CDX-2 (green), CAFs by tagging α-SMA (red), exogenous CAFs by tagging mCherry (violet), and nuclei by tagging DAPI (blue). Some cells that were positive for α-SMA in the lung metastases of mice in the HCT-116+MSC group were also positive for mCherry, which suggested that some CAFs in the lung metastases originated from the primary tumor (Fig. 2C).

**Figure 4.**
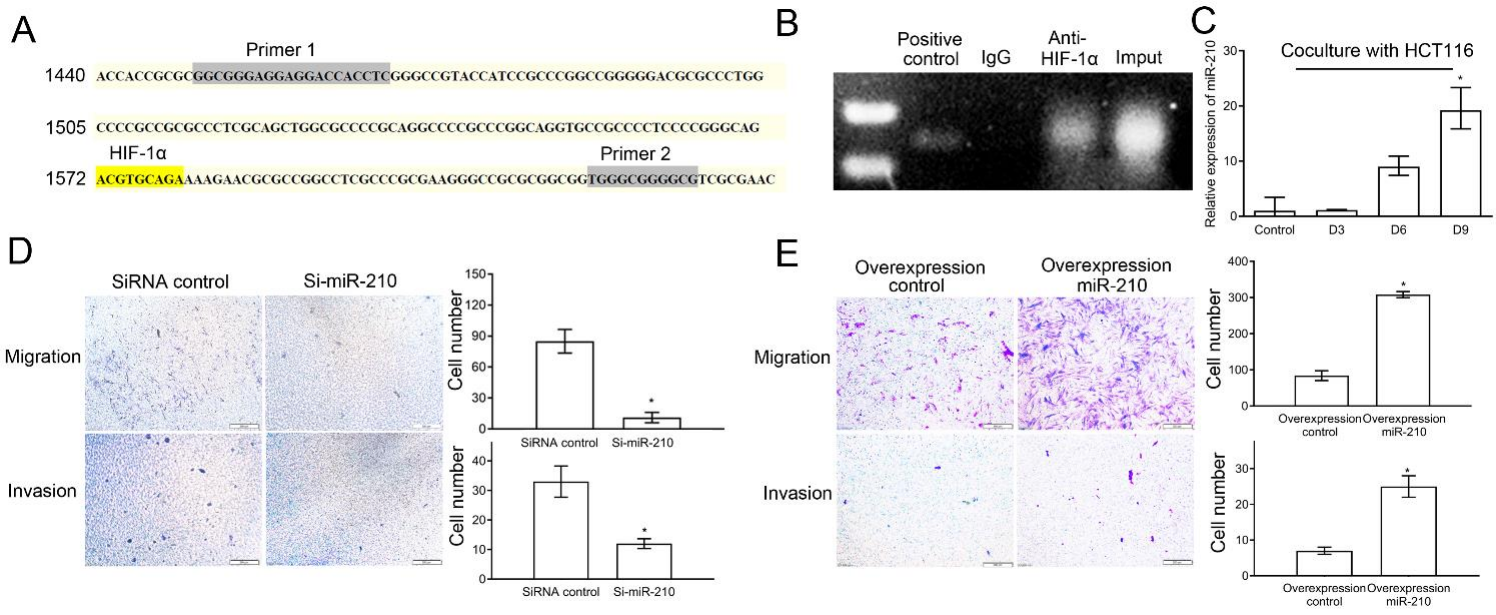
HIF-1α regulated the migration and invasion of CAFs by upregulating the expression of miR-210. (A) Binding sites of HIF-1α in the miR-210 promoter region. (B) ChIP assay revealed that HIF-1α could bind to the miR-210 promoter region. (C) qRT-PCR showing that the expression of miR-210 was upregulated during the differentiation of MSCs into CAFs induced by HCT-116 cells. * *P* < 0.05. (D) Transwell assays (n = 4) revealed that the migration and invasion of CAFs decreased significantly after interference with miR-210 in CAFs. * *P* < 0.05. Scale bar: 200μm. (E) Transwell assays (n = 4) revealed that the migration and invasion of CAFs increased significantly after miR-210 overexpression in MSCs. * *P* < 0.05. Scale bar: 200μm.

### HIF-1α regulated the migration and invasion of CAFs

In contrast to normal fibroblasts, CAFs play a role in metabolic reprogramming, and HIF-1α is an important regulatory factor of this process [29]. HIF-1α regulates tumour cell migration and invasion. Therefore, we aimed to investigate whether HIF-1α affected the migration and invasion functions of CAFs. The results showed that the expression of HIF-1α was upregulated during the differentiation of MSCs into CAFs (Fig. 3A), and HIF-1α was significantly increased in the nucleus (Fig. 3B). We further found that the migration and invasion capacities of the induced cells were significantly decreased when the HIF-1α inhibitor KC7F2 (S7946, Selleck, 10 μmol/l) was added to the conditioned medium (Fig. 3C).

### HIF-1α regulated the migration and invasion of CAFs by upregulating the expression of miR-210

Bioinformatics analysis revealed a binding site for HIF-1α in the promoter region of miR-210 (Fig. 4A). We performed a ChIP assay and found that HIF-1α could bind to the miR-210 promoter region in CAFs (Fig. 4B). Moreover, the expression of miR-210 was gradually upregulated during the differentiation of MSCs into CAFs (Fig. 4C). In addition, the overexpression of miR-210 in CAFs was decreased significantly by the protein inhibitor for HIF-1α (KC7F2, 10 μmol/l) in the conditioned medium (Supplementary Fig. 4). We subsequently interfered with the expression of miR-210 in MSCs (see Supplementary Fig. 5A for interference efficiency). HCT-116 cells were then used to induce the differentiation of Si-miR-210-MSCs into CAFs. The migration and invasion abilities of CAFs in which miR-210 expression was altered were significantly reduced compared with those of the control group (Fig. 4D). However, the migration and invasion capacities of CAFs were significantly upregulated when miR-210 was overexpressed in MSCs (Fig. 4E, see Supplementary Fig. 5B for overexpression efficiency).

**Figure 5.**
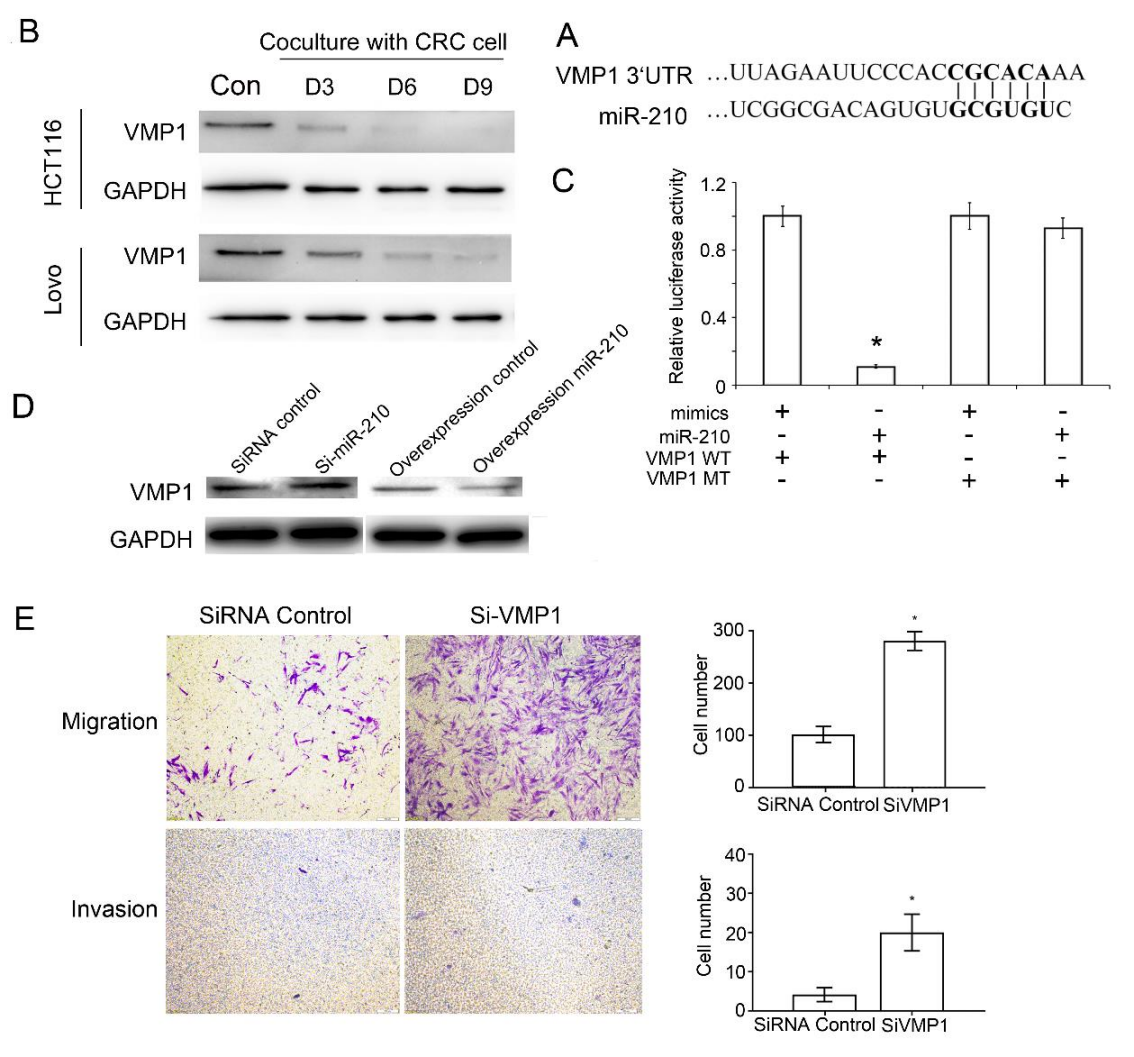
miR-210 regulated the migration and invasion of CAFs by regulating VMP1. (A) Bioinformatics analysis showing that miR-210 could directly bind to the 3’UTR of VMP1. (B) Western blot showing the downregulated expression of the VMP1 protein during the differentiation of MSCs into CAFs. (C) Dual-luciferase reporter assay suggested that miR-210 could bind to the 3’UTR of VMP1 (VMP1 WT: VMP1 3’UTR Wild type; VMP1 MT: VMP1 3’UTR mutation type; mimic: microRNA control). * *P* < 0.05. (D) Western blot analysis showing the upregulated and downregulated expression of the VMP1 protein after interference with and overexpression of miR-210, respectively. (E) Transwell assays (n = 4) showing that the migration and invasion of CAFs significantly increased after interference with VMP1. * *P* < 0.05. Scale bar: 200μm.

### miR-210 regulated the migration and invasion of CAFs by downregulating the expression of VMP1

As a non-coding RNA, the biological effect of miR-210 depends on its interaction with mRNA. According to bioinformatics analysis, miR-210 has a binding site in the 3’UTR of VMP1, Bcl-2, FOBX31 and HIF3α [30-32] (Fig. 5A, Supplementary Fig. 6A). However, only VMP1 expression was significantly downregulated during the differentiation of MSCs into CAFs induced by colorectal cancer cells (Fig. 5B, Supplementary Fig. 6B). The dual-luciferase reporter assay showed that miR-210 could directly bind to the 3’UTR of VMP1 (Fig. 5C). Interference with miR-210 expression resulted in the upregulation of VMP1 expression during the differentiation of MSCs into CAFs, while overexpression of miR-210 resulted in the downregulation of VMP1 expression (Fig. 5D, Supplementary Fig. 6C). Interference with VMP1 expression increased the migration and invasion capabilities during the differentiation of MSCs into CAFs (Fig. 5E, see Supplementary Fig. 5C for interference efficiancy). To further confirm that miR-210/VMP1 regulated the migration and invasion of CAFs, a rescue experiment was conducted by overexpressing miR-210 and/or VMP1 and revealed that the ability of miR-210 to upregulate the migration and invasion of CAFs could be impaired by overexpressing VMP1 (Supplementary Fig. 6D).

**Figure 6.**
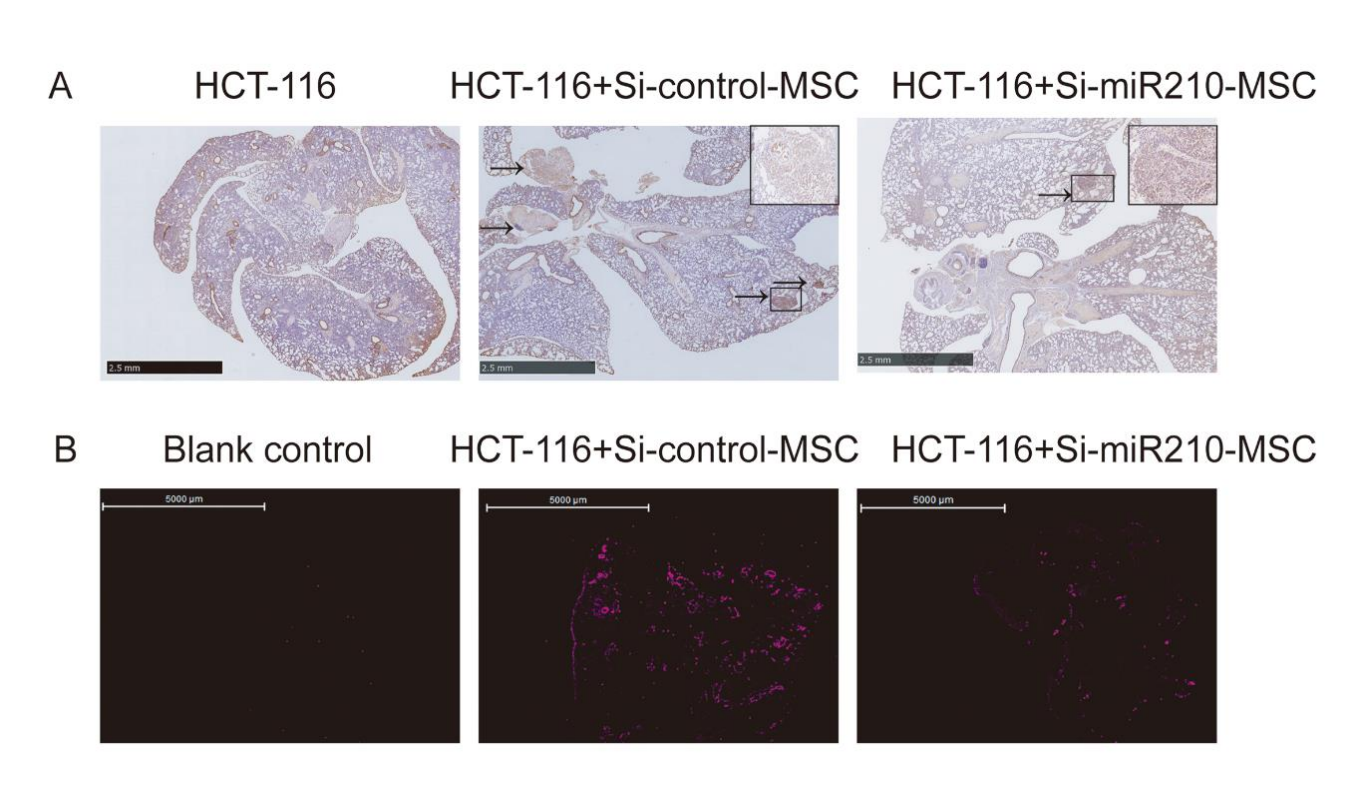
Validation of the effect of the HIF-1α/MIR-210 pathway on the metastasis of cancer cells and CAFs. The metastasis of cancer cells and CAFs was detected in a subcutaneous xenograft model of colorectal cancer. Lung metastases of colorectal cancer cells (arrows in A, CDX-2 IHC, scale bar: 2.5 mm.) and exogenous CAFs (tagged violet mCherry in B, scale bar: 5000 μm.) were reduced after interference with the expression of miR-210 in the subcutaneously transplanted MSCs.

### Interfering with the expression of miR-210 in CAFs reduced both CAFs and CRC lung metastases in mice

We further verified the effect of the HIF-1α/miR-210 pathway on the metastasis of cancer cells and CAFs in the process of MSCs being induced into CAFs in the subcutaneous xenograft model of CRC. The results showed that the lung metastasis of cancer cells in the HCT-116 combined with MSCs group was significantly more than that of the HCT-116 alone group (*p* = 0.019, Fig. 6A, Supplementary Fig. 7A). However, the metastasis-promoting effect of CAFs was weakened significantly after interfering with the expression of miR-210 (*p* = 0.04, Fig. 6A, Supplementary Fig. 7A). In addition, we found that the ability of CAFs to metastasize to the lungs was also impaired after interfering with the expression of miR-210 (Fig. 6B, Supplementary Fig. 7B-7C).

## DISCUSSION

In the present study, we found that CAFs differentiated from MSCs in the subcutaneous xenograft model of CRC could metastasize from the primary site to distant organs and promote tumor metastasis. We further proved that the enhanced migration and invasion abilities of CAFs might be caused by the upregulation of HIF-1α expression, which promoted the transcription of miR-210, thereby inhibiting the expression of the VMP1 protein (Supplementary Fig. 8).

MSCs are one of the main sources of CAFs in tumour tissues. We also confirmed that MSCs could differentiate into CAFs after being induced by CRC cells both in vitro and in vivo. Normal MSCs have limited migration ability, but almost no invasion ability [33, 34]. Previous studies suggested that CAFs were more migratory than normal fibroblasts in lung cancer [35]. Our study also showed that the migration and invasion abilities of CAFs were significantly higher than those of MSCs.

We then verified that CAFs could promote tumor metastasis in the subcutaneous xenograft model of CRC, which is consistent with the results of Wen *et al* in breast cancer [36]. Further analysis of CAFs at pulmonary metastases revealed that some of the CAFs were anthropogenic, which means that they had metastasized from the primary tumor. Duda *et al* showed that cancer cells brought their own soil, CAFs, to metastasize together, which promoted the survival and colonization of circulating cancer cells in a lung cancer metastasis model [14]. Circulating CAFs could also be distinguished in the peripheral blood of patients with breast cancer, prostate cancer, colorectal cancer and lung cancer. In these studies [15, 16], the level of cCAF was associated with clinical metastasis and could be a maker for the predication of metastasis. And the circulating CAFs levels also correlated with worse prognosis and a shorter survival time in metastatic cancer patients. All these results indicate that CAFs in the primary tumor can metastasize and promote tumor metastasis. Tumor metastasis is the main cause of death in CRC patients, and inhibition of CAF metastases may theoretically help to reduce tumor metastasis, so we further studied the possible mechanism by which CAFs regulate metastasis.

Hypoxia is the most important feature of tumors and plays a very important role in the processes of tumor proliferation and metastasis. Hypoxia induced HIF-1α expression in tumor cells, followed by metabolic reprogramming, angiogenesis, stromal remodeling, and migration and invasion enhancement [37]. Previous studies have shown that hypoxia or high HIF-1α expression could directly (ZEBI, Snail, Twist, CAV-1) or indirectly (Notch, TGF-β, integrin-linked kinase) regulate EMT and the migration and invasion of various tumour cells [38-40]. Similar to tumor cells, the HIF-1α signaling pathway also has the ability to regulate the migration of MSCs [41-43]. In addition, a previous study suggested that hypoxia could stimulate a small number of MSCs to enter the circulating blood [44]. Since the tumor microenvironment is also hypoxic, we speculated that HIF-1α might play an important role in the tumor microenvironment and regulate the invasion and migration of CAFs. Our study found that HIF-1α expression in CAFs was upregulated compared with that in MSCs. Similarly, the expression of HIF-1α was also activated when fibroblasts were induced to CAFs after co-culture with breast cancer cells in previous studies [45, 46]. After HIF-1α inhibition, we found that the invasion and migration of CAFs decreased significantly, indicating that hypoxia also plays a key role in the invasion and migration of CAFs.

HIF-1α is an important transcription factor. Among the genes that HIF-1α or hypoxia regulates transcriptionally, miR-210 has been shown to regulate the migration and invasion (metastasis) of a variety of tumors, especially CRC [47-50]. Although miR-210 has been proven to be a target of HIF-1α in breast cancer cells [51], whether HIF-1α regulates miR-210 transcription in CAFs has not yet been reported. In our study, we found that overexpression of miR-210 in MSCs significantly increased the migration and invasion of induced CAFs, while inhibition of miR-210 expression in CAFs reduced migration and invasion. These results suggested that miR-210 was also related to the regulation of invasion and migration in CAFs. We then proved through ChIP experiments that HIF-1α could directly bind to the promoter region of miR-210 in CAFs. In addition, the expression of miR-210 decreased after HIF-1α inhibition in CAFs, suggesting that HIF-1α promotes the migration and invasion of CAFs by regulating the transcription of miR-210. In the mouse xenograft model, the numbers of metastatic foci and human CAFs in the lungs were reduced after inhibiting the expression of miR-210 in MSCs transplanted subcutaneously together with colon cancer cells. Therefore, miR-210 might be a good target for inhibiting CAF metastasis. There are relatively few studies on the mechanism of CAF migration and invasion. We found for the first time that the mechanism by which hypoxia promotes metastasis in tumor cells can also be applied to CAFs, providing a new understanding of CAFs.

miR-210 is a non-coding RNA that normally suppresses downstream gene expression by targeting the 3’UTR of mRNA. We predicted the target genes of miR-210 on a bioinformatics website. Combined with a literature search, we identified several possible downstream targets of miR-210 related to migration and invasion, such as Bcl-2, HIF3α, and VMP1 [30-32]. Vacuolar membrane protein 1 (VMP1) is considered to be an important protein in cancer. VMP1 was initially reported to regulate tumor cell migration and invasion in a liver cancer study [52]. The expression of VMP1 was significantly decreased in CRC tissues compared with adjacent non-cancer tissues [53]. Therefore, VMP1 is considered to play a vital role in the process of tumor metastasis. Moreover, miR-210 can bind to the 3’UTR of VMP1 and regulate the invasion and migration of tumor cells [32, 54, 55]. Since the metastasis of CAFs is similar to that of tumor cells, we hypothesized that the invasion and migration of CAFs in the tumor microenvironment were also regulated by VMP1. Finally, we confirmed that VMP1 was the downstream target of miR-210 and regulated the invasion and migration of CAFs.

Considering the important role of CAFs in tumor proliferation, metastasis and drug resistance, CAF-related treatment seems to be a good direction. The results of previous studies suggested that tumor metastasis could be inhibited by hindering the formation and functions of CAFs in vitro [35] [56]. In the present study, we reported the possible mechanism by which CAFs metastasize in CRC and further confirmed in vivo that inhibiting the migration and invasion of CAFs could inhibit metastases of both tumors and CAFs. These results are innovative. However, there are still some limitations to our research. First, we did not confirm the correlation between the amount of circulating CAFs and prognosis in CRC patients. This may require collecting peripheral blood from CRC patients and separating cCAFs for further analysis. Second, the mechanism by which CAFs colonize themselves and then help tumor cells colonize and survive in distant target organs needs to be further studied.

In summary, our study revealed that upregulation of the HIF-1α/miR-210 pathway can enhance the migration and invasion of CAFs, and CAFs from the primary foci of CRC can metastasize to distant organs and promote tumor metastasis. These results provide new insights for future related research and new ideas for tumor treatment.

## Supplementary Materials

The Supplemenantry data can be found online at: www.aginganddisease.org/EN/10.14336/AD.2021.0315.


