# Supplementary material for: HIF-1α promotes the migration and invasion of cancer-associated fibroblasts by miR-210
Source: Aging Dis. 2021 Oct 1;12(7):1794–807. doi: 10.14336/AD.2021.0315 (PMC8460292; doi:10.14336/AD.2021.0315)
Supplement: Supplementary file 1 [file AD-12-7-1794-s.pdf]

SUPPLEMENTARY DATA

HIF-1α promotes the migration and invasion of cancer-associated fibroblasts by miR-210

Ying Yang[1], Junjie Gu[1], Xuechun Li[2], Chunling Xue[2], Li Ba[2], Yang Gao[1], Jianfeng Zhou[1*], Chunmei Bai[1*], Zhao Sun[1*,] Robert Chunhua Zhao[2*]

© 2021. Yang Y et al. Published online at http://www.aginganddisease.org/EN/10.14336/AD.2021.0315

SUPPLEMENTARY DATA

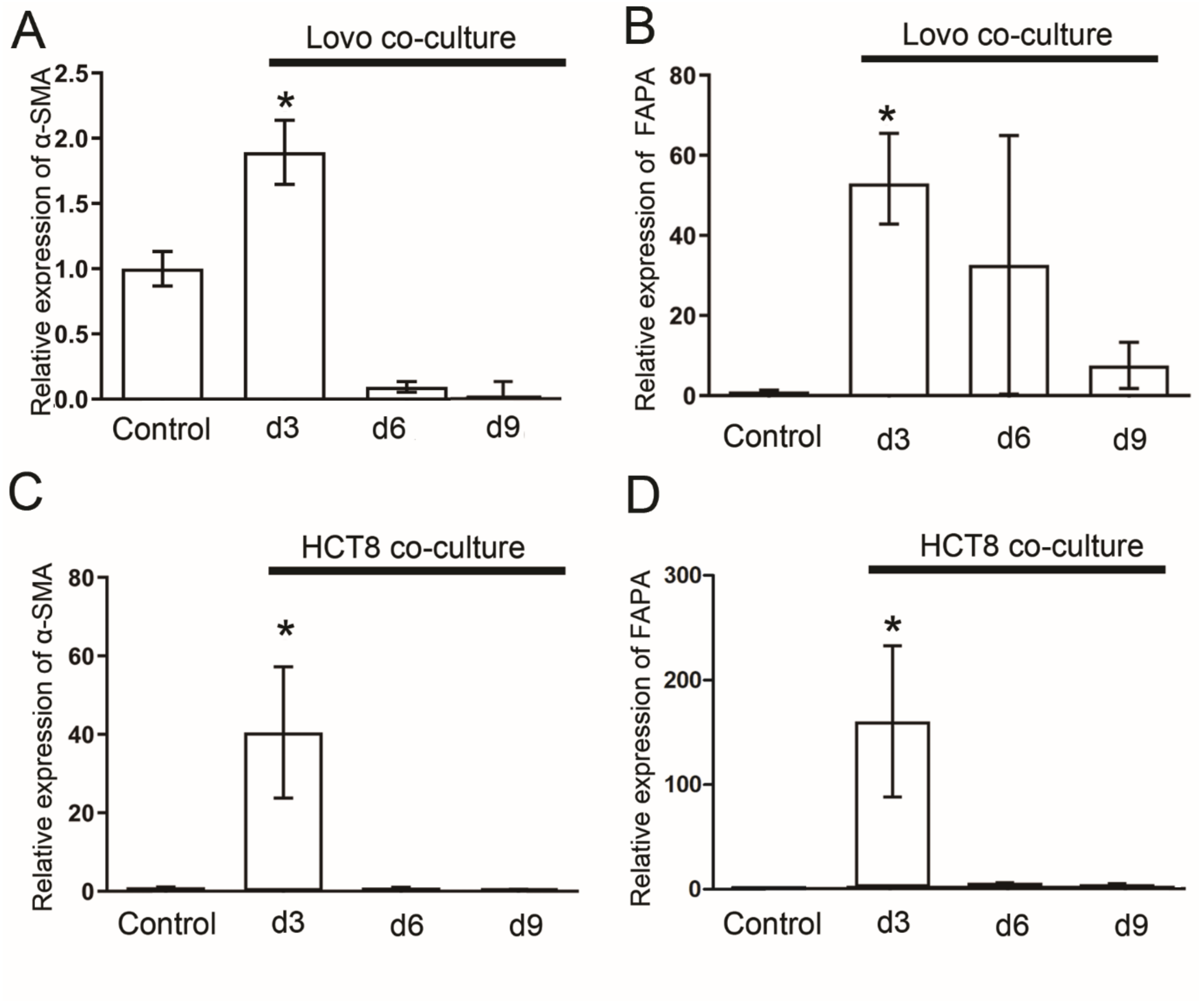

Supplementary Figure 1. The expression of characteristic genes in CAFs was detected by qRT-PCR. * $P < 0.05$

© 2021. Yang Y et al. Published online at http://www.aginganddisease.org/EN/10.14336/AD.2021.0315

SUPPLEMENTARY DATA

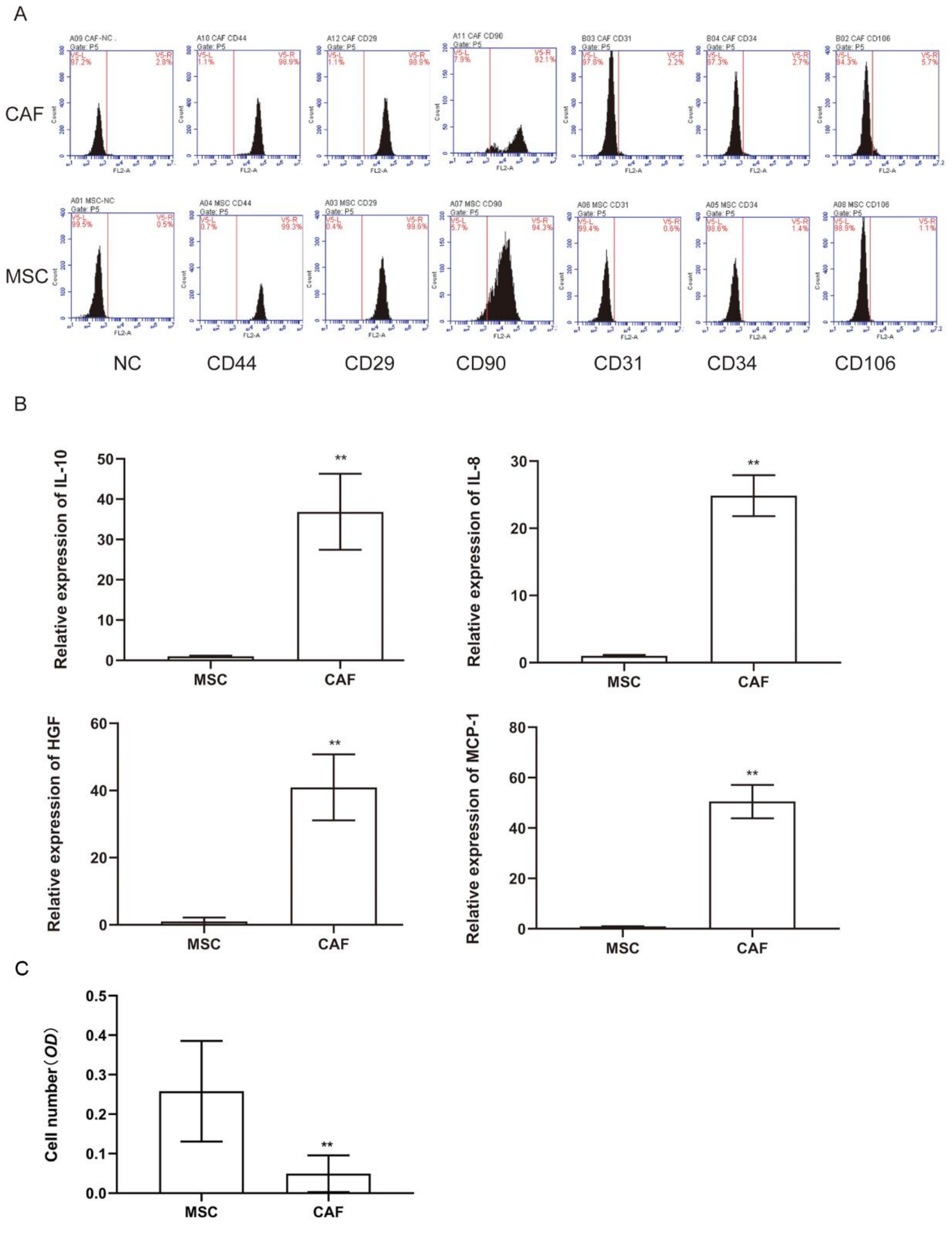

Supplementary Figure 2. Comparison of the characteristics of MSCs and CAFs. (A) Flow cytometry identification of mesenchymal markers. **(B)** qRT-PCR was used to detect the expression of IL-8, IL-10, MCP-1, and HGF of MSCs and CAFs. **(C)** Adhesion assay was used to detect the adhesive capacity of MSCs and CAFs. ** $P < 0.01$

SUPPLEMENTARY DATA

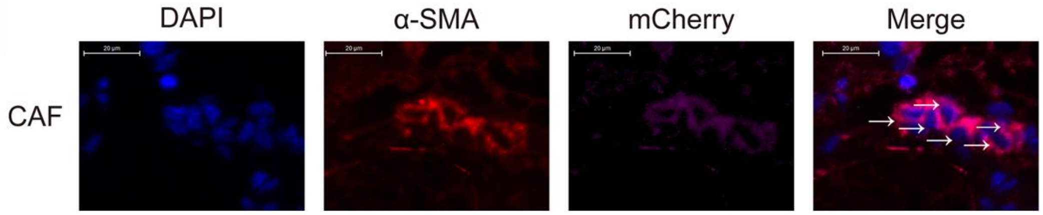

Supplementary Figure 3. Multiple immunofluorescence staining detecting the exogenous CAFs. Exogenous MSCs could differentiate into CAFs (white arrows) in the primary tumors of subcutaneously implanted colorectal cancer models. DAPI: nucleus (blue), α-SMA: CAFs (red), mCherry: exogenous MSCs (violet). Scale bar: 20 μm.

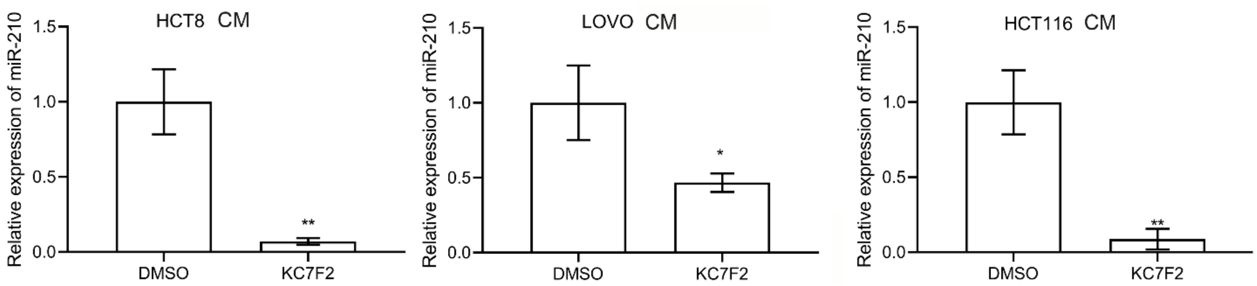

Supplementary Figure 4. miR-210 expression was downregulated after HIF-1α was inhibited. The culture medium of HCT-8, HCT-116 or LOVO cells was collected as the conditioned medium (CM). MSCs were cultured with conditioned medium in a six-well plate. DMSO was added to the control group, and KC7F2 (S7946, Selleck), an HIF-1α selective protein translation inhibitor, was added to the experimental group. * $P < 0.05$; ** $P < 0.01$.

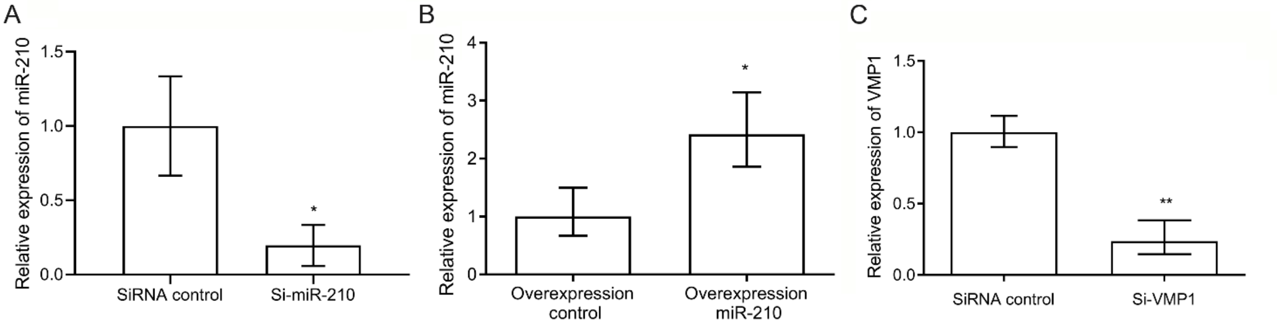

Supplementary Figure 5. qRT-PCR was used to detect transfection efficiency. (A) The interference efficiency of miR-210. * $P < 0.05$. **(B)** The overexpression efficiency of miR-210. * $P < 0.05$. **(C)** The interference efficiency of VMP1. ** $P < 0.01$.

© 2021. Yang Y et al. Published online at http://www.aginganddisease.org/EN/10.14336/AD.2021.0315

SUPPLEMENTARY DATA

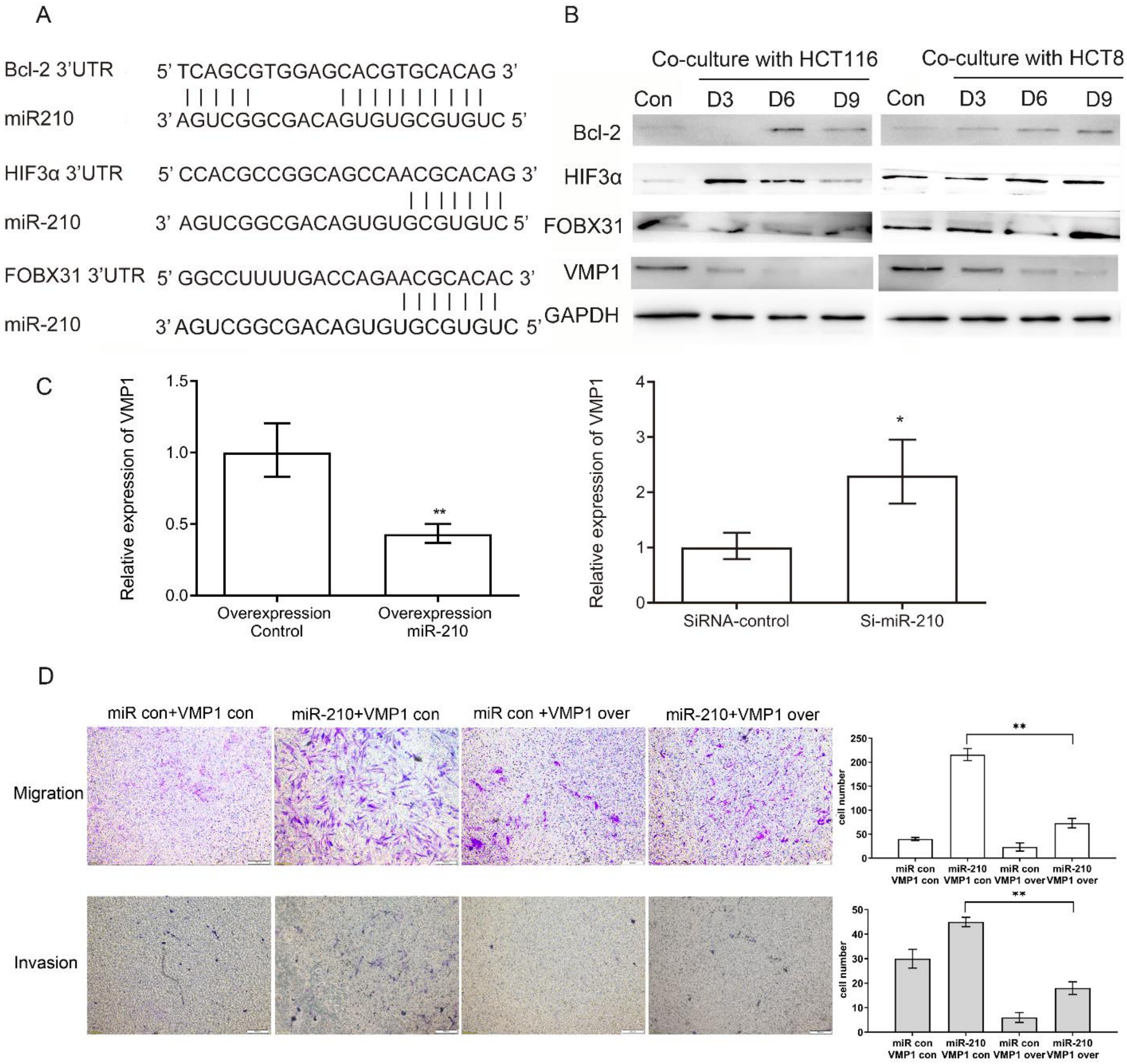

Supplementary Figure 6. miR-210 regulated the migration and invasion of CAFs by downregulating the expression of VMP1. (A) Bioinformatics analysis showing that miR-210 could directly bind to the 3'UTR of Bcl-2, HIF3α, and FOBX31**. (B)** Western blot showing no decrease in the expression of the Bcl-2, HIF3α, and FOBX31 protein during the differentiation of MSCs into CAFs. **(C)** qRT-PCR was used to detect the mRNA expression of VMP1 after miR-210 interference or overexpression. * $P < 0.05$; ** $P < 0.01$. **(D)** VMP1 overexpression rescued the promoting effects of miR-210 overexpression on CAF migration and invasion. miR con+VMP1 con indicates the control group. miR-210+VMP1 con indicates that only miR-210 was overexpressed. miR con+VMP1 over indicates that only VMP1 was overexpressed. miR-210+VMP1 over indicates that both miR-210 and VMP1 were overexpressed. n = 4. ** $P < 0.01$. Scale bar: 200μm.

© 2021. Yang Y et al. Published online at http://www.aginganddisease.org/EN/10.14336/AD.2021.0315

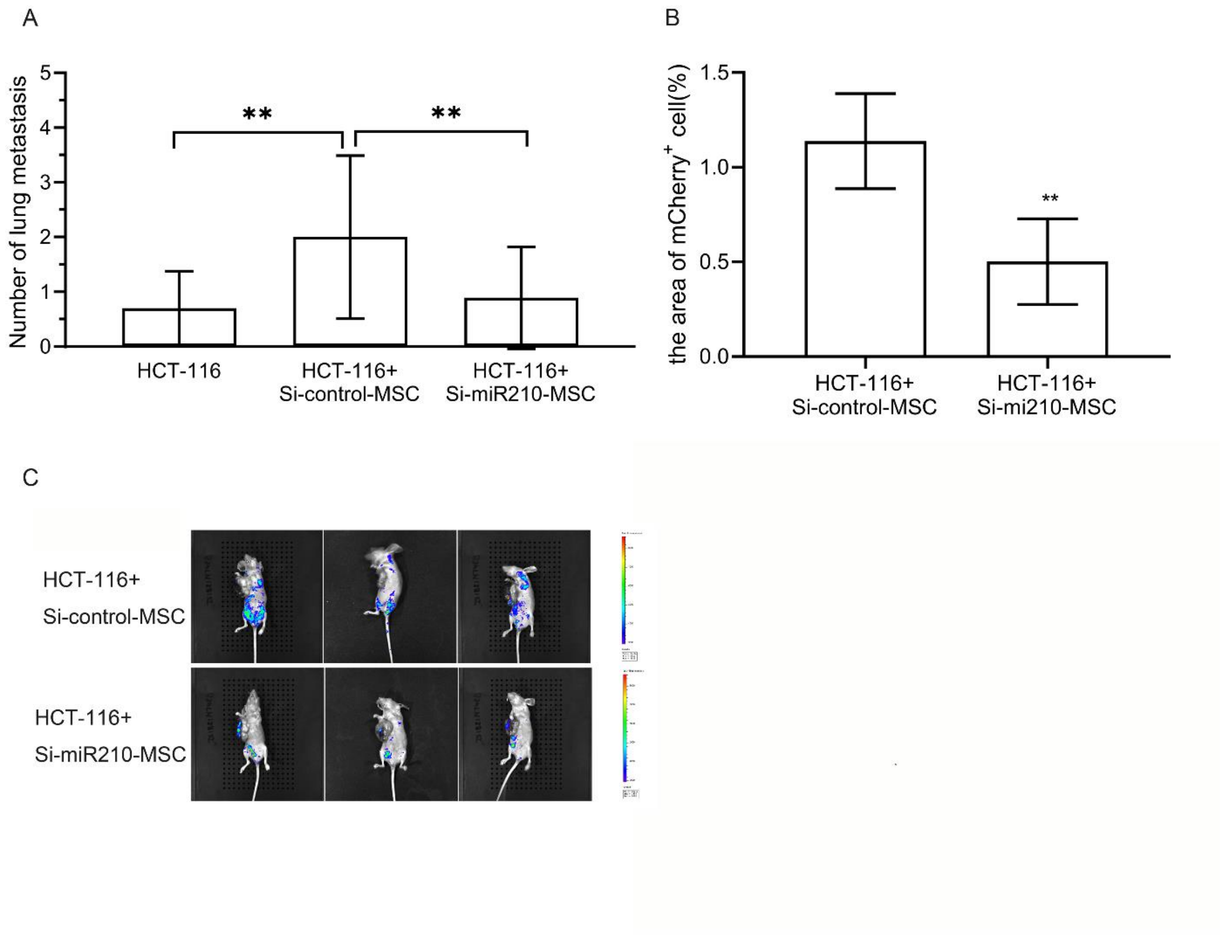

Supplementary Figure 7. Validation of the effect of the HIF-1α/miR-210 pathway on the lung metastasis of CRC and CAFs. (A) The number of CRC lung metastasis in each group**. (B)** The area of mCherry^{+} cells in each group. n = 10. * $P < 0.05$, ** $P < 0.01$. **(C)** In vivo fluorescence image showing CAFs metastasis in the subcutaneous transplantation model. The CAFs metastasis was reduced after interference with the expression of miR-210.

© 2021. Yang Y et al. Published online at http://www.aginganddisease.org/EN/10.14336/AD.2021.0315

SUPPLEMENTARY DATA

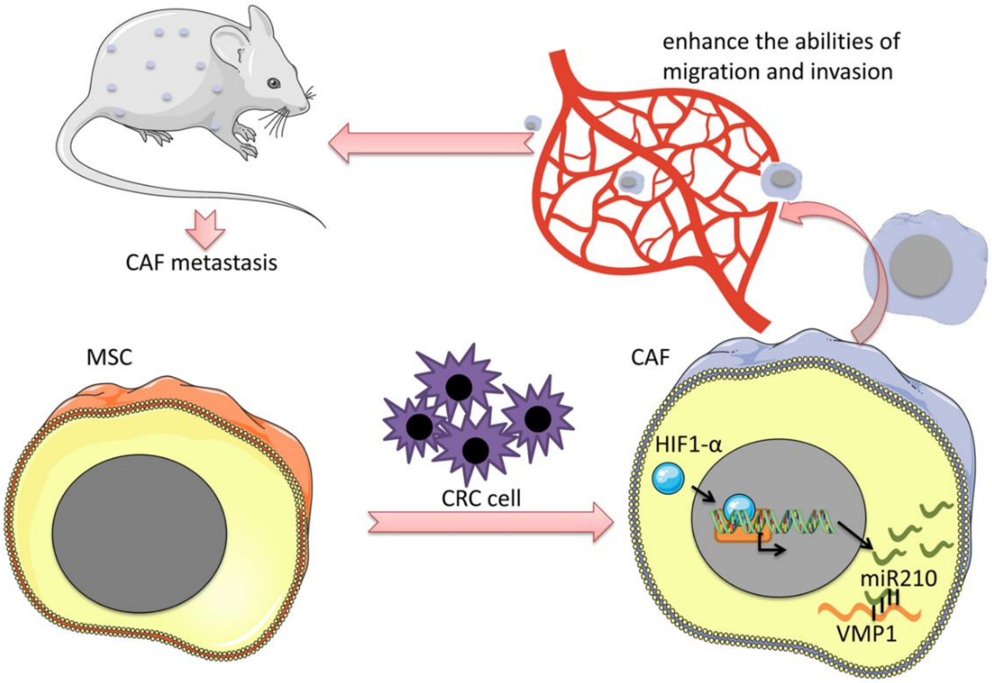

Supplementary Figure 8. Schematic overview of the HIF1-α/miR210/VMP1 in the regulation of the migration and invasion in CAFs

Supplementary Table 1. The primer sequences.

U6	forward primer	CTCGCTTCGGCAGCACATATACT
	reverse primer	ACGCTTCACGAATTTGCGTGTC
GAPDH	forward primer	GGTCACCAGGGCTGCTTTTA
	reverse primer	GGATCTCGCTCCTGGAAGATG
miR-210	forward primer	CTGGAGCTGTGCGTGTGACAGC
	reverse primer	GTGCAGGGTCCGAGGT
VMP1	forward primer	GAGATGCTGGAACATGCAGA
	reverse primer	TTGCTCCACTATGTGCTTGC
IL-8	forward primer	ACTCCAAACCTTTCCACCCC
	reverse primer	TTCTCAGCCCTCTTCAAAAACTTC
IL-10	forward primer	GTGGCATTCAAGGAGTACCTC
	reverse primer	TGATGGCCTTCGATTCTGGATT
MCP-1	forward primer	CAGCCAGATGCAATCAATGCC
	reverse primer	TGGAATCCTGAACCCACTTCT
HGF	forward primer	TCT TGA CCC TGA CAC CCC
	reverse primer	GTG ATT CAG CCC CAT CCG G

© 2021. Yang Y et al. Published online at http://www.aginganddisease.org/EN/10.14336/AD.2021.0315
